# Aortic Dissection Associated With Seizures

**DOI:** 10.7759/cureus.43116

**Published:** 2023-08-08

**Authors:** Lamiz Tannouri, Shahinaz Gouda, Lazima Cholasseri

**Affiliations:** 1 Emergency Medicine, Soliman Fakeeh University Hospital, Dubai, ARE; 2 General Practice, Soliman Fakeeh University Hospital, Dubai, ARE

**Keywords:** aortic diseases, neurologic symptoms, chest pain, seizures, aortic dissection

## Abstract

We present a case of an extensive aortic dissection identified in a young male patient with atypical symptoms. The patient presented to the Emergency Department with severe sudden onset chest pain and seizure. In the Emergency Department, bedside Transthoracic Echocardiography (TTE) identified moderate pericardial effusion which correlated with a widened mediastinum on chest radiograph. The presence of aortic dissection was confirmed by an urgent CT angiogram of the thorax and abdomen. Our case highlights the importance of critical thinking in the atypical presentation of aortic dissection in which this diagnosis may not be considered, leading to negative and possibly deadly outcomes.

## Introduction

Aortic dissection is a life-threatening cardiovascular emergency characterized by a tear in the inner lining of the aorta, resulting in the formation of a false lumen. Aortic dissection is commonly associated with hypertension, atherosclerosis, and advancing age. However aortic dissection occurring in young individuals, especially males is rare and poses significant diagnostic and management challenges. 

This case study aims to present a comprehensive analysis of a young male patient with aortic dissection, highlighting the unique clinical features, rapid diagnosis, treatment approach, and long-term outcomes associated with this condition. The case involves a 34-year-old male, with no known comorbidities, who presented to the emergency department with severe central chest pain without radiation and a first episode of seizure. 

Overall, this case study aims to demonstrate the challenges associated with atypical presentation of aortic dissection in young otherwise healthy patients, involving neurological features. And highlight how rare neurological presentations, such as seizures, may mislead the diagnosis. 

## Case presentation

A 34-year-old male with previously no known comorbidities, presented to the Emergency Department (ED) with central chest pain for about 3 hours. He was brought by ambulance with a history of severe chest pain that started at 1 pm, the pain was followed by an episode of seizure. As per the patient, he woke up from an afternoon sleep with pain and sweating, the pain was in the center of the chest without radiation. A witness reported that the patient collapsed and began to shake uncontrollably throughout their body during the episode, with uprolling of the eyes and frothing around the mouth. The episode of jerking lasted for less than five minutes and abated without medication. In the ambulance, the patient's blood pressure (BP) was 80/50 mmHg, heart rate (HR) 124 beats per minute, and for his active pain, he received aspirin on the way. Upon arrival, the patient was immediately shifted to the resuscitation room, as he was agitated and complaining of severe chest pain. 

**Figure 1:**
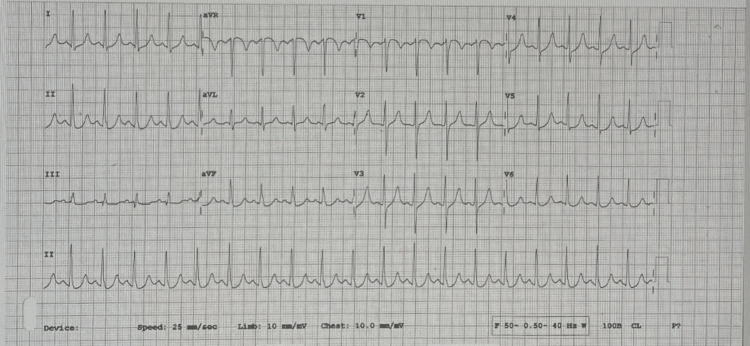
ECG done in the ED ECG showing sinus tachycardia, with upsloping ST depressions on inferior leads and lateral leads (DII, AVF and V4 -V5) with mild AVR elevation.

On primary assessment, the patient was conscious and oriented, in active pain, and diaphoretic. BP was equal in both arms. Pulses were equal in all limbs. The chest examination was clear, with good air entry bilaterally. Neurologic examination was unremarkable. Electrocardiogram was promptly done (Figure 1) showing sinus tachycardia, with upsloping ST depressions on inferior and lateral leads (DII, AVF, and V4 -V5) with mild AVR elevation. 

**Figure 2:**
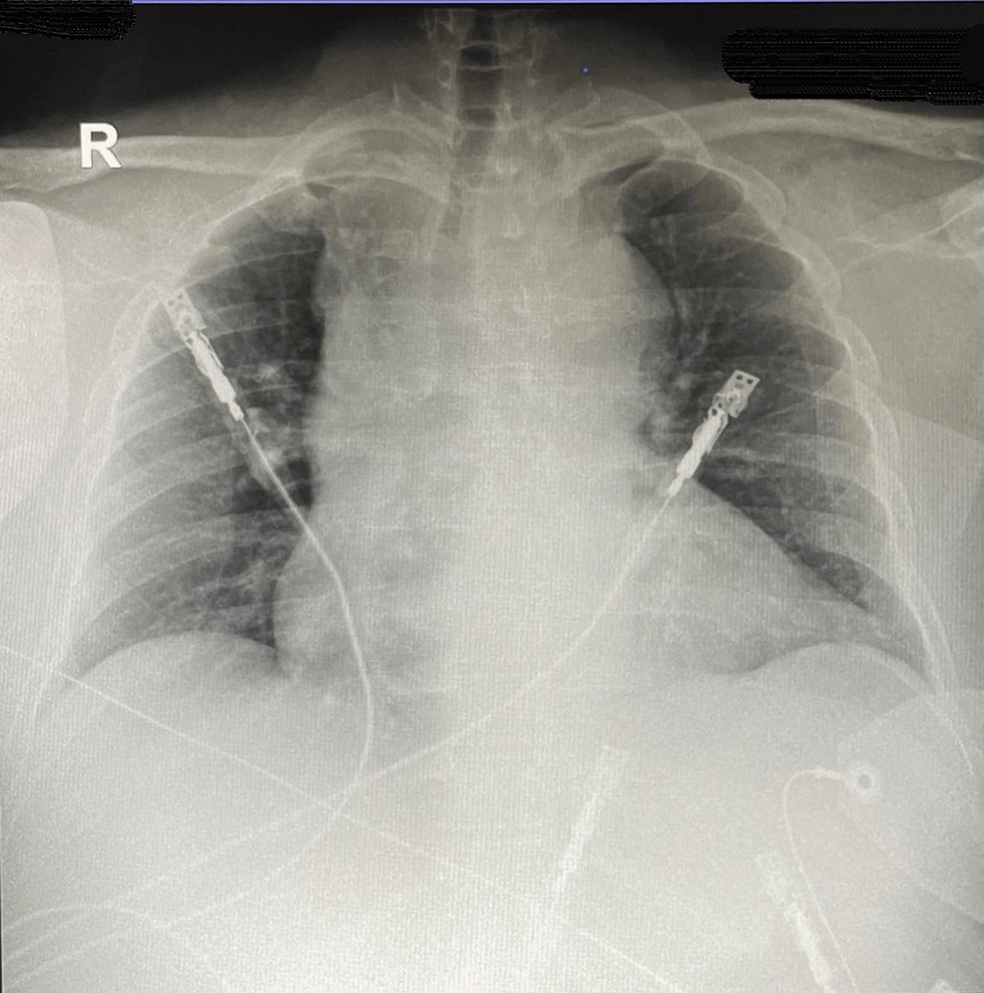
Portable chest X-ray showing generalized cardiomegaly with mediastinal widening

Initial management by the emergency team was promptly started. In the ER, the patient received intravenous fluids (IVF), and analgesia, and was kept on a monitor with defibrillator pads. The pain was controlled and BP improved to 86/75 mmHg after IVF. Cardiology was consulted who performed a transthoracic echocardiogram (TEE), which showed global hypokinesia, severe left ventricular hypertrophy, and moderate pericardial effusion. The calculated Ejection Fraction (EF) was 55%. No tamponade or aortic flap was visualized. A portable chest X-ray (Figure 2) was done which showed generalized cardiomegaly with mediastinal widening. 

**Figure 3:**
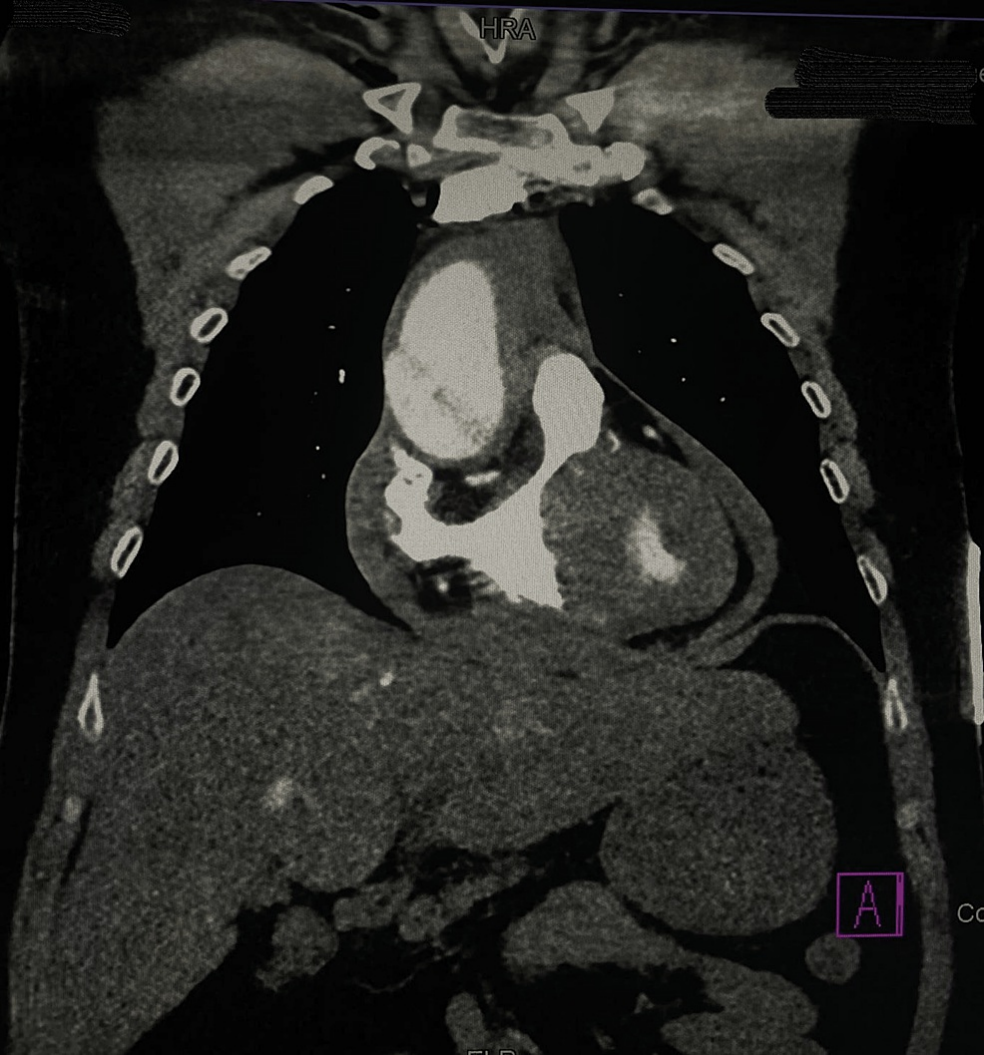
CT Angiography of Chest Computed tomography (CT) angiography of the chest showed ascending aortic ectasia, Type A, with focal rupture of the false lumen resulting in moderate pericardial effusion and mediastinum hematoma, the dissection flap raised 2 cm from the aortic valve. The coronary arteries arise from the true lumen.

**Figure 4:**
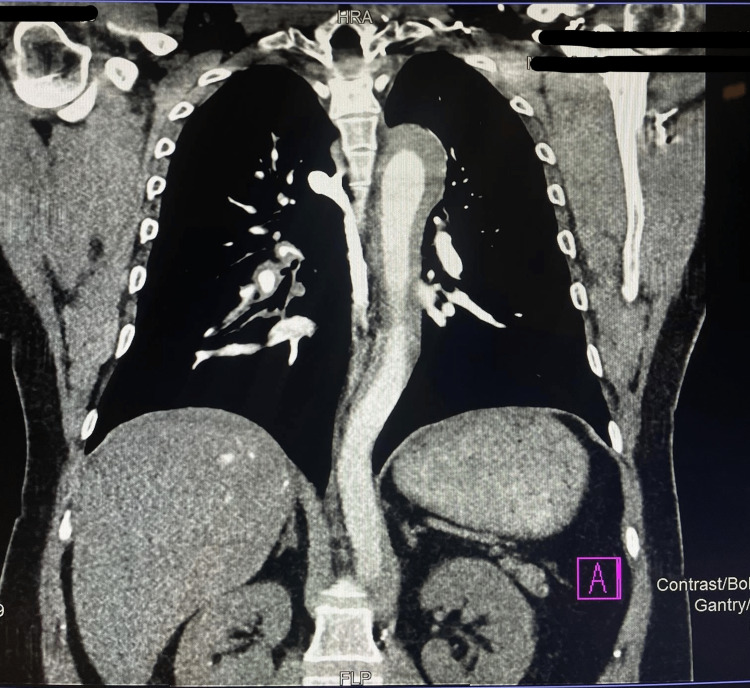
CT angiography of the chest showing intramural hematoma thrombosed on the descending thoracic aorta

**Figure 5:**
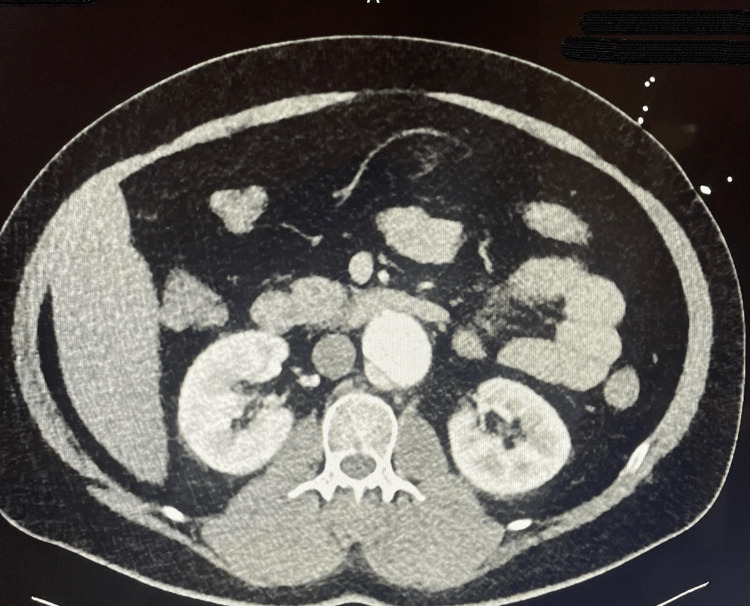
CT angiography of the abdomen showing that a second dissection flap was noted distal to the superior mesenteric artery.

The possibility of aortic dissection was considered and the patient's BP was kept stable with a mean arterial pressure (MAP) above 65 with medical management. He was taken for an immediate Computed Tomography (CT) angiogram of the chest and abdomen (Figures 3, 4, 5). CT showed ascending aortic ectasia Type A, with focal rupture of the false lumen resulting in moderate pericardial effusion and mediastinum hematoma, the dissection flap raised 2 cm from the aortic valve. A second dissection flap was noted distal to the superior mesenteric artery, extending to the infrarenal aorta, aortic bifurcation, common iliac arteries, and proximal left internal iliac artery. Additionally, a thrombosed intramural hematoma was noted in the descending thoracic aorta. The coronary arteries arise from the true lumen. There was no evidence of extension of the dissection into major neck arteries.

**Figure 6:**
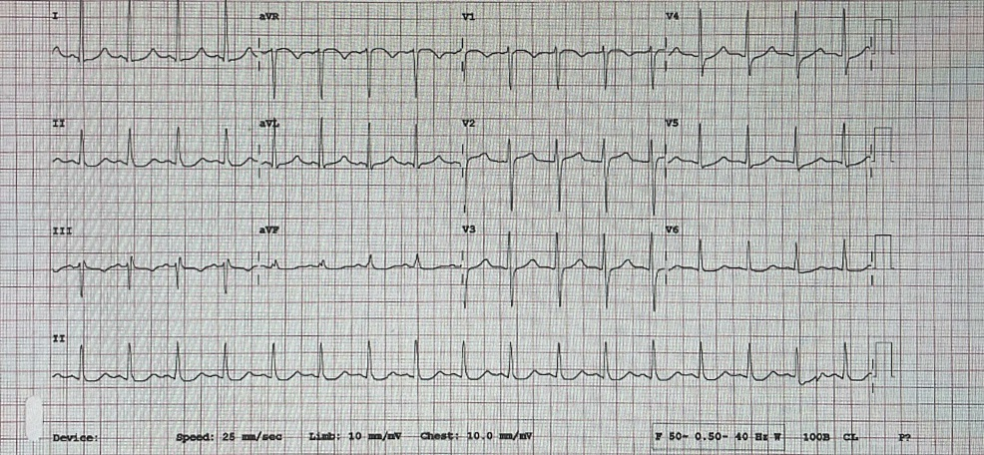
Post-procedure ECG showed improvement from the previous ischemic changes

Intensive care service and cardiothoracic were contacted. The patient was immediately sent to the operating theater. Aortic dissection repair was done with ascending aortic graft and the patient was admitted to the ICU post-procedure. Post-procedure ECG showed improvement from the previous ischemic changes (Figure 6). He was discharged after one week without any neurological deficits and in stable condition. He followed up with a cardiologist and cardiothoracic surgeon later. 

## Discussion

The annual incidence of aortic dissection varies between 5 and 30 per million people. Aortic dissection is an uncommon but serious condition that can be fatal. Most patients die before reaching the hospital or receiving a diagnosis [1,2]. It’s essential to keep a high clinical index of suspicion for this condition to perform a rapid and accurate diagnosis [1,2]. Studies suggest that 38% of patients don't receive the diagnosis of acute aortic dissection at the time of onset [1,3].

Numerous studies have shown a higher prevalence among men compared to women, the prevalence in men ranges from two to five times compared to women. The average age of proximal dissection was between 50 to 55 years old [1,2]. Multiple studies have shown that 62% to 78% of cases of aortic dissection are associated with chronic systemic hypertension as the most common predisposing factor [1,2].

Even though pain is the most common symptom related to aortic dissection, more than one-third of the patients present with secondary symptoms related to other organ systems involvement. Neurological symptoms in aortic dissection instances are frequently transient, fluctuating, and completely resolved before arrival to the emergency room. These signs are typically observed at the start of the dissection process or shortly thereafter. The fast improvement of the neurologic status could be correlated to the transient arterial occlusion at the time of the dissection [1,2].

In the case series presented by Gaul at el., ischemic stroke symptoms were the most common initial findings related to neurological manifestation. Among the 102 patients analyzed with aortic dissection, 30 patients (29%) exhibited initial neurological symptoms. Out of these patients, only two-thirds reported experiencing chest pain. The neurological symptoms were subsequently linked to various conditions, including ischemic stroke (16%), spinal cord ischemia (1%), ischemic neuropathy (11%), hypoxic encephalopathy (2%), and syncope (6%) [1,2]. It is worth noting that seizures were observed in only 3% of these patients [2,4]. On presentation, our patient presented neurologically intact, with no signs of focal weakness or slurred speech, completely conscious and oriented. The first episode of seizure was his only neurological manifestation documented.

Finsterer et al. reviewed a case involving an 88-year-old woman who experienced a first episode of a generalized tonic-clonic seizure. After successfully managing the seizures, the patient reported experiencing cramping pain and involuntary flexion movements in the right upper extremity. She was initially vitally stable, however, her blood pressure deteriorated with time. Magnetic resonance imaging (MRI) of the brain did not show ischemia or intracerebral bleeding. The presence of pericardial effusion was observed during transthoracic echocardiography. The aortic dissection type A diagnosis was confirmed with a CT angiography of the thorax. The cardiothoracic team declined to perform surgical treatment due to the advanced age of the patient and the high risks, and the patient died 16 hours after admission [4]. The authors of this case report proposed that one could speculate on the possibility that the dissection initially resulted in general cerebral hypoperfusion, leading to hypoxia or stenosis of the common carotid artery, as well as cerebral ischemia. However, these ischemic effects were not apparent on the patient's brain MRI [4]. Similarly, our patient did not show any signs of hypoxia or hypoperfusion during presentation, which would have indicated the occurrence of an ischemic event.

Patients with aortic dissections that extends into the carotid arteries are more likely to exhibit neurological symptoms. These symptoms may include stroke-like symptoms, altered mental status, or syncope. Rivera-Alvarez et al. described a case of a 49-year-old male, known hypertensive, dyslipidemic, and prediabetic who was brought to ED unconscious. During the primary assessment, it was observed that the individual was unresponsive, with stiff arms, extended legs, clenched jaw, and foam in his mouth. The CT angiography of the chest revealed a Stanford type A aortic dissection that extended into the left proximal subclavian artery, as well as a 6 cm aortic root aneurysm. The dissection also involved the great vessels, including the brachiocephalic, left common carotid, and left subclavian arteries [5]. 

Another case report published by Eikelboom et al. described a 57-year-old woman status epilepticus. The CT angiography of the patient's chest and abdomen revealed a type A aortic dissection that extended from the coronary sinuses to the right common iliac artery. Additionally, it was observed that the right common carotid artery was occluded, which likely contributed to the patient's neurological status [3].

We presented a case of a 34-year-old male with chest pain and seizure as the first presentation. TEE showed moderate pericardial effusion and a CT angiogram detected type A aortic dissection. The patient's BP was borderline during his stay in the ED. After the seizure, the patient's neurological examination was unremarkable, he was fully conscious and oriented without any deficits. The patient was taken to urgent surgery and was discharged without complications. As highlighted in the above discussion, seizure at first presentation is uncommon, affecting only 3 % of the cases described in the literature. It was also described that most neurological presentations are correlated with the dissection extending to the carotid arteries. Despite the neurological event, no carotid dissection or occlusion was detected in our patient. 

## Conclusions

Aortic dissection is an uncommon and fatal condition. In rare occasions, it can present as syncope, seizure, or altered mental status. The literature review highlighted that very few cases of neurological presentations of aortic dissection were published, and even fewer presented with seizures as the first symptom. Most of the neurological presentations showed proximal extension of the dissection to the carotid and vertebral arteries. Our case showed a young male with seizure and chest pain. Unlike the previously published cases, his CT did not show any proximal extension of the dissection. ED physicians should keep a high clinical index of suspicion to diagnose aortic dissection, as this condition has a variety of presentations and needs rapid diagnosis and fast management. 
